# Characteristics of patients with organic brain syndromes : A cross-sectional 2-year follow-up study in Kuala Lumpur, Malaysia

**DOI:** 10.1186/1744-859X-4-9

**Published:** 2005-04-15

**Authors:** Prem K Chandrasekaran, Stephen T Jambunathan, Nor Z Zainal

**Affiliations:** 1 NeuroBehavioural Medicine, Penang Adventist Hospital, Penang, Malaysia; 2 Department of Psychological Medicine, University of Malaya Medical Centre, Kuala Lumpur, Malaysia

## Abstract

**Background:**

Organic Brain Syndromes (OBS) are often missed in clinical practice. Determining their varied presentations may help in earlier detection, better management, and, assessing prognosis and outcome. We described the in-patient referrals of patients suffering from the psychiatric effects of organic states and compared the symptomatology and mortality between those with the Acute and Chronic varieties.

**Methods:**

59 patients referred to our Consultation-Liaison (C-L) Psychiatry services and given a clinical diagnosis of OBS were selected over a 6-month period. Psychiatric and cognitive abnormalities and treatment regimes were recorded and fatality rates determined. Information regarding their condition 24 months after their index hospitalization was recorded. All data were entered into a proforma and analyzed after exclusion.

**Results:**

The mean duration of detecting the symptoms by the physician was 3.52 days. The presence of a premorbid psychiatric illness had no influence on the clinical presentation but did on the mortality of patients with OBS **(p = 0.029)**.

Patients with the Acute syndrome had significantly more symptom resolution as compared to those with the Chronic syndrome **(p = 0.001) **but mortalityrates did not differ. Elderly patients and those with symptom resolution upon discharge did not show statistically significant higher mortality rates. The most popular combination of treatment was that of a low-dose neuroleptic and a benzodiazepine (34.7%). The need for maintenance treatment was not significantly different in any group, even in those with a past history of a functional disorder.

**Conclusion:**

Other than the Acute group having a significantly better outcome in terms of symptom resolution, our findings suggest that there was no significant difference in the clinical presentation between those with Acute or Chronic OBS. Mortality-wise, there was also no difference between the Acute and Chronic syndromes, nor was there any difference between the elderly and the younger group. There was also no significant difference in the need for continued treatment in both groups.

## Background

Diseases of the brain are frequently manifested by psychiatric symptomatology, a condition conventionally termed 'Organic Brain Syndrome'. Given the complexity of the nervous system and the vast range of pathological processes that can affect it, a broader view that there exist a number of different and distinct organic brain syndromes seems more likely. OBS is not a specific neurological diagnosis although it remains a standard diagnostic category. One justification for the use of the term is as a kind of abbreviated phrase to refer to the full range of abnormal mental symptoms commonly associated with definable neurological disease [1]. It should be stressed that OBS are defined in psychiatric terms and not in neurologic terms. They are purely descriptive and carry no specific aetiologic implications [2]. Considering the variety of pathological processes that fall under this heading, it is not surprising that no one particular agent has proven to be of significant benefit to date [1]. Symptoms suggestive of cognitive impairment may even persist in a proportion of cases long after the initial episode, especially when the cerebral insult is irreversible [3]. The aims of this study were:

(1) to measure the efficiency of medical personnel in detecting patients suffering from the psychiatric effects of organic states,

(2) to compare the various patterns of clinical presentation between those with the Acute and Chronic varieties of OBS,

(3) to assess the mortality of these neuropsychiatric episodes after a 2 year period, and,

(4) to determine the various ways psychotropic medications were used

## Methods

### Sample

A total of 196 patients were referred to the C-L Psychiatry services of the Department of Psychological Medicine, University of Malaya Medical Centre (UMMC) between 1^st ^March and 30^th ^September, 1998. Of this number, 59 patients were diagnosed to have OBS and this sample constituted the focus of this study. Being a cross-sectional follow-up study, the 3 patients whose case notes were not traceable were excluded from the sample.

### Materials

The data were collected from the referral records and further information was obtained from the patient's case notes. Based on the case notes, all cases were assessed during the index admission by a Trainee Psychiatrist, the Principal Investigator (PI i.e. author **PK**), and a Consultant Psychiatrist within 3 hours of receiving the referral form. Patients who were diagnosed to have Acute or Chronic OBS were selected for this study. Their demographic data, psychiatric history (which included clinical presentation and premorbid personality), medical history, mental status examination, physical examination, laboratory investigations, treatment and the progress, in terms of symptom resolution, were recorded. The data were used for specific sub-diagnoses according to the Diagnostic and Statistical Manual of Mental Disorders – 4^th ^Edition (DSM-4) [4] and the fatality rates were determined.

Determining the number of days that elapsed from the onset of symptoms to the time the C-L referral was made gave a crude assessment of efficiency of medical personnel in detecting OBS. The case notes were examined further to see the follow-up progress of patients after 2 years. The PI then called the patients who had defaulted follow-up for enquiries about their condition and treatment. All data entered into the proforma was validated by the Lecturer involved, the Second Investigator (author **ST**).

### Statistical analysis

With the Consultant involved, the Third Investigator (author **NZ**), overseeing the progress, the data were analyzed using the Statistical Package for the Social Sciences (SPSS) 7.5. Descriptive statistics were presented as mean plus or minus standard deviation (SD) and the differences between groups were assessed by the independent samples t-test for equality of means (2-tailed).

Categorical data were analyzed using the Pearson's Chi-square test (2-sided) for differences between the Acute and Chronic groups or the Fisher's exact test (2-sided), where appropriate. The level of significance is **p = < 0.05**.

## Results

### A. Demographic data

44 of the total number of patients were below the age of 65 (78.6%) and 12 were above 65 (21.4%). 37 were male (66.1%) and 19 were female (33.9%).

### B. Descriptive data

#### 1) Duration of symptoms before referral

The minimum number of days elapsed from onset of symptoms to the C-L referral was 0 days and the maximum was 16 days. The mean value was 3.52 days and the SD was 3.29.

#### 2) Underlying psychiatric disorder

Listed below are 17 of the 49 patients who had premorbid psychiatric illnesses and all of them with functional diagnoses were in remission at the time of this study.

• Alzheimer's dementia – 5

• Major depression – 3

• Alcoholic dementia – 2

• Post-ictal psychosis – 1

• Alcoholic hallucinosis – 1

• Mental retardation with Bipolar affective disorder – 1

• Post-concussional dementia – 1

• Brief reactive psychosis – 1

• SLE-induced psychosis – 1

• Simple deteriorative disorder – 1

#### 3) Perceptual disturbances and thought disorder

17 of the patients (30.4%) experienced visual hallucinations, 15 (26.8%) of them had auditory hallucinations and only 12 (21.4%) were deluded.

#### 4) Cognitive functions

17 patients had global disorientation to time, place and person. Furthermore, all those disorientated had disorientation to time. All patients had impairment of attention and concentration (Table 1).

**Table 1:** Cognitive functions

**Disorientation to:**	**Frequency**	**%**
Time	48	85.7
Place	25	44.6
Person	23	41.1

**Impairment of:**	Frequency	**%**

Recent memory	54	96.4
Remote memory	41	73.2
Attention and concentration	56	100.0

#### 5) Liaison psychiatry diagnosis

A clear organic triggering factor could be found for all patients. 49 (87.5%) of them had Acute OBS and only 7 (12.5%) had the Chronic variety. The respective coded DSM-4 diagnoses, with specific coding, were given (Table 2).

**Table 2:** Liaison psychiatry diagnosis

Acute:
**293.0 **– There were 44 with delirium due to various causes:
• Head trauma – 6
• Uremia – 4
• Post-ictal state – 4
• Post-operative state – 2
• Brain metastasis – 2
• Hyperglycaemia – 2
• Burn trauma – 2
• Anaemia – 2
• Cerebral infarction – 2 (1 with alcohol-induced persisting dementia – **291.2**)
• Hepatic encephalopathy – 1
• Septicaemia – 1
• Multiple myeloma – 1
• Cerebral lupus – 1
• Cerebral hypoxia – 1
• Hyponatremia – 1 (with co-existing thyrotoxicosis)
**291.0 **– Alcohol withdrawal delirium – 6 (1 with co-existing delirium due to hypoglycaemia – **293.0**)
**292.81 **– Steroid-withdrawal delirium – 2
**290.11 **– Dementia of Alzheimer's type, early onset, with delirium due to post- operative state – 1
**290.11 **– Dementia of Alzheimer's type, early onset, with delirium due to non- convulsive status – 1
**290.3 **– Dementia of Alzheimer's type, late onset, with delirium due to carcinoma – 1
**292.81 **– Opioid intoxication delirium – 1
**293.81 **– Psychotic disorder due to Cushing's disease, with delusions – 1
**293.82 **– Psychotic disorder due to end stage renal failure – 1
**293.83 **– Mood disorder due to acute myocardial infarction – 1
**293.83 **– Mood disorder due to post-operative state – 1
**293.83 **– Mood disorder due to cerebral lupus – 1

**Chronic:**

**290.40 **– Uncomplicated vascular dementia – 2
**290.42 **– Vascular dementia with delusions – 1
**290.43 **– Vascular dementia with depressed mood – 1
**290.20 **– Dementia of Alzheimer's type, late onset, with delusions – 1
**290.0 **– Dementia of Alzheimer's type, late onset, uncomplicated – 1
**294.0 **– Alcohol-induced amnestic disorder, chronic – 1

#### 6) Psychiatric treatment

**Figure 1:**
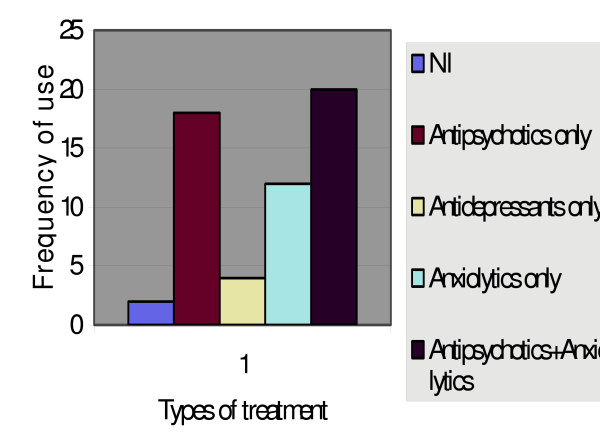
**Psychiatric treatment**. The category axis (y) represents the types of treatment used and the value axis (x) represents the frequency of use.

Only 2 did not require psychiatric treatment and they were those with vascular dementia and morphine intoxication delirium. Of the 54 that required it, 21 of them required relatively high doses i.e. 12 with delirious states, including all 5 alcohol withdrawals, 2 with vascular dementias, 1 with the organic psychotic disorder and 1 with the organic mood syndrome. 3 of them had a previous history of a mental illness. Only 9 of them were agitated. 32 of the 49 (65.3%) with acute syndromes required relatively low doses of medication. 27 of the 33 (81.8%) that required low doses were in a delirium (Figure 1).

#### 7) Total symptom resolution (upon discharge)

34 (60.7%) of the total had symptom resolution on discharge and 22 of them (39.3%) did not. Below is the breakdown of symptom resolution for the specific subgroups.

• Delirium – 33 of the 45 (73.3%) had total symptom resolution

• Dementia – 5 of the 10 (50.0%) with dementia had no symptom resolution

• Organic psychotic disorder – 1 with post-ictal state and 1 with Cushing's disease had symptom resolution

• Organic mood syndrome – only the 1 with post-operative state recovered

• Transient amnestic disorder – the 1 with this disorder had total resolution of symptoms

#### 8) Mortality

19 of these patients (33.9%) had passed away during the 2-year period and another 19 had defaulted follow-up. There were only 18 (33.9%) alive at the end of this study.

#### 9) Continuing treatment

At the end of this study period, of the 18 that could be traced, 14 were not on treatment and of the 4 who were still on treatment, 1 of them was in the Chronic group – vascular dementia – and 3 in the Acute group – cerebral hypoxic delirium, organic psychotic disorder and organic mood syndrome. 3 of them had a past history of a mental disorder and all of them were on Chlorpromazine, Thioridazine, Sulpiride or Risperidone. 3 had been on Haloperidol and 1 on Mianserin during the index admission.

### C. Difference in clinical presentation between those with the Acute and Chronic varieties

None of these analyses proved to be of any significance.

### D. Influence of previous psychiatric history on hallucinations and delusions in OBS

Again, none of these associations proved significant. Cross-tabulations reported p = 0.919, p = 0.770, p = 0.336 respectively for visual hallucinations, auditory hallucinations and delusions.

### E. Association between psychiatric diagnosis and symptom resolution upon discharge

Those patients with the Acute syndrome had significant symptom resolution as compared with those having the Chronic syndrome **(p = 0.001)**. However, the elderly patients had no significant decline towards symptom resolution as compared to the younger age group (p = 0.127).

### F. Presence of previous psychiatric history and symptom resolution upon discharge

In 1 patient with OBS and a history of a mental illness, symptom resolution after commencing treatment was not prolonged as indicated by an index of p = 0.167.

### G. Effect of psychiatric diagnosis and mortality

There was no difference in terms of mortality between those with the Acute or Chronic varieties of OBS. Even in older patients with OBS, a value of p = 0.124 showed that there was no significant difference in mortality as compared to those younger than 65 years old.

### H. The association between symptom resolution upon discharge and mortality

There was no significant association between these 2 variables.

### I. Influence of presence of previous psychiatric history in those with OBS on mortality

This association proved to be of statistical significance **(p = 0.029) **indicating that patients with a premorbid mental disorder had lower mortality rates.

### J. The need for continued treatment in the subgroups of OBS

This was not significant (p = 0.405) showing that those with the Chronic syndrome required no more maintenance treatment as compared with the Acute group. And in those with a previous psychiatric history, the need for maintenance treatment was no different from those without (p = 0.275). Even in the elderly patients, there was no increased need for continued treatment, as evidenced by a value of p = 0.405.

## Discussion

Medical records provide a useful source of information and diagnoses based on medical records are acceptable as long as they are considered a substitute of diagnoses obtained from a direct interview. Telephone interviewing is also considered an acceptable alternative method and it has been reported that comparable diagnostic information is obtained through face-to-face and telephone interviews [5]. We had used both modalities to a certain extent and they had their limitations, as would be discussed later.

In this study, the geriatric group made up less than a quarter of the sample, and on the whole, males predominated the sample by two-thirds. The mean duration of time elapsed from onset of symptoms in comparison with the SD proved that detection of these syndromes has been rather inefficient in this center (3.52 days). The association between elapsed time and symptom resolution was not significant. Although almost a third of them had a previous history of a mental illness, it had no bearing on the presence of hallucinations and delusions, nor did it on symptom resolution or the need for continued treatment. Those with the previous history did, however, require higher doses of medication as compared to the rest because of their underlying psychiatric illness. Oddly, there were significantly lower mortality rates (p = 0.029) in those who had a previous history of psychiatric disorder, possible reasons being that those cases may not have been OBS in the first place but misdiagnosed instead, and also the small number in that category. The above findings suggest that premorbid functional disorders do not affect the clinical presentation of patients during the course of an OBS. However, since all the patients with premorbid mental illnesses involved in the study were in remission, the above suggestion cannot be concluded.

When there are severe perceptual disturbances in the visual modality, acute cerebral disorder is more implicated than the chronic type [6]. Visual hallucinations predominated the clinical picture in contrast to auditory hallucinations and delusions, but again did not vary in their occurrence between both varieties of OBS. In a study by Hirono [7], it was found that half of their patients with Alzheimer's disease showed evidence of delusions or hallucinations. Independent factors associated with psychosis were older age, female sex, longer duration of illness and more severe cognitive impairment. Orientation to time is labile and quickly disrupted by organic causes. Orientation to place is disturbed later in the disease process. When established, disorientation to time and place are evidence of an organic state and may be the earliest signs in a dementing process. Disorientation to person occurs at a very late stage. It was found that a very high number of patients experienced disorientation to time and less than half were disorientated to place and person. This points to the early detection of these cases before their condition deteriorated and produced global disorientation. Memory disturbances associated with brain disease is referred as organic or true amnesia and manifest as impairments of registration, retention, retrieval, recall and recognition. In organic states, attention may be profoundly decreased and usually accompanied by lowering of consciousness [8]. Almost all patients had impairment of recent memory and only just over a quarter of them had remote memory impairment. Attention and concentration was, however, impaired in all of them.

We tackled the confusion surrounding the Acute-Chronic dichotomy by carrying on the initial diagnosis given by the PI and Consultant Psychiatrist during the index admission and going by the possible reversibility of a particular condition instead of the rapidity of its development or resolution. Put simply, the primary cause of the acute impairment is usually 'outside the brain' and that of the chronic syndrome normally 'within the brain'. The distinction between these two organic conditions is most clearly derived from the history of the mode of onset of the disorder. A short history and firm knowledge of an acute onset will make a chronic reaction unlikely and onset in association with a physical illness is strongly suggestive of an acute organic reaction [6]. The use of specific diagnoses is helpful as although most chronic organic disorders cannot be reversed, a small number are potentially treatable [9]. Acute disturbances of cerebral function may, in time, progress to the development of irreversible structural pathology with an admixture of features specific to both. The two may co-exist when a chronic dementing process is complicated by another concomitant or superimposed disease [6]. Those with delirium superimposed on dementia were designated as Acute as their symptoms in their index admission were those of a delirious nature. As expected, it was found that symptom resolution occurred with significance in the Acute group as compared to the Chronic group (p = 0.001). However, the younger age group did not show any statistical significance toward symptom resolution as compared to the older group. Delirium has poor outcomes in hospitalized older patients [10]. It has multiple aetiologies and a poor long-term prognosis [11]. Advancing age increases the risk and those over 60 years are at highest [12]. The older the patient and the longer the delirium, the outcome is a longer resolution of symptoms. A complete resolution of confusional symptoms is not usually achievable in prolonged confusional states that are superimposed on dementia. Improvement from severe to mild confusion or merely a reduction of symptoms would be a more realistic goal [13]. However, in this study, it was found that those who had delirium on dementia had resolution of their confusional symptoms. Even with treatment, there was no improvement in their dementing features, as may be expected.

It was found that there was no significant difference in mortality rates between the Acute and Chronic groups, possibly due to the small number of patients assigned to the latter group. This was in keeping with observations made by Inovye [14] that there were no significant associations between delirium and mortality and between delirium and length of hospital stay. That study, however, found delirium to be a significant predictor of functional decline at both hospital discharge and at follow-up, therefore making it an important independent prognostic determinant of hospitalization outcomes. Our findings disagreed with the generally held concept that the occurrence of delirium was associated with a high mortality rate in the following year, mainly because of the serious nature of the provoking medical conditions. Even the mortality in the elderly within the sample showed no statistical significance as compared to those who were younger than 65 and the finding was not in keeping with related literature. Huang [15] investigated the rate of delirium, reasons for admission, clinical features, aetiologies and mortality during a 2-year follow-up and found that the incidence of delirium was higher in their geriatric group. However, the older patients had a higher mortality rate during the 2-year follow-up period and that stressed the importance of after-discharge care in those patients. Higher death rates have also been found among the cognitively impaired elderly patients than those aged-matched patients with functional psychiatric illnesses and the cognitively intact elderly. Koponen[16] was of the same school of thought and associated delirium with a significant rate of mortality. These results, however, were not in line with findings by Rabins & Folstein [17] that cognitively impaired individuals have higher fatality rates than cognitively intact individuals. There was also no significant association between symptom resolution upon discharge during the index admission and mortality.

As observed earlier, the most popularly used treatment in our setting was a combination of a neuroleptic and a benzodiazepine, usually Haloperidol and Lorazepam. This combination accounted for the treatment of over a third of patients and the use of a neuroleptic alone came second, amounting to just under a third of the patients. Adams [18] showed that parenteral Haloperidol offered the first hope for treating delirium and the addition of Lorazepam quickened the onset of sedation. Delirium is a common component of dementia and may produce considerable morbidity. In addition to psychotic features, it may produce considerable agitation, which may be unresponsive to conventional medications. The main approach is to treat any underlying medical condition that could cause the delirium. It is, however, not always reversible and there is no specific treatment for persistent delirium [19]. Cole, Primeau and Elie [20] found Haloperidol, Chlorpromazine and Mianserin to be useful in controlling the symptoms of delirium and high levels of premorbid functioning were related to better outcomes. The use of this selection of drugs was similarly practiced in our setting although Chlorpromazine is now less widely used and usually reserved more for its sedative-hypnotic effects. This is because we have had experiences with its propensity to lower the seizure threshold and to cause hypotension. Finally, there was no significant difference in the need for continued treatment at 2 years in the Chronic group as compared to the Acute group. Even in those with a previous psychiatric history or in those who were in the elderly age group, there appeared to be no difference.

Only 15 patients afflicted with these conditions were compliant to follow-ups. There were only another 3 of those who defaulted follow-up and whose conditions were documented in their case notes when they were subsequently admitted for other problems unrelated to that of their index admission. Thus, there were still 19 of them whose status at 2 years was unknown. The problem was mainly with those having alcohol-related disorders and it has been found that patients with alcohol delirium have been known to have higher mortalities and have been known to be more difficult to follow-up [16]. Of the 8 with these disorders, 2 had passed away, 4 were not contactable and the 2 that were eventually contacted had not turned up for follow-ups. This large number of dropouts where follow-ups were concerned caused difficulties in assessing the mortality rate after 2 years. It proves to be a major issue in C-L Psychiatry and needs to be addressed to ensure more comprehensive post-discharge care to this group of patients.

Although the methods by which data were obtained in this study have been validated previously [17], the questionable reliability of the data collected from the medical records forms the first limitation. There was also little information on the outcome of these patients in the records and as earlier mentioned, telephone calls revealed no new information. The second limitation was that assessment scales had not been incorporated. Another limitation to this study was the small sample size and confined only to the UMMC thus, we were not able to apply the results as representing a whole region. Also, the relatively small number of patients with a diagnosis that suited the criteria for the Chronic syndrome had caused difficulties in statistical analysis, as did the high rate of dropouts on establishing the mortality rate after 2 years. This study was intended to promote practical awareness and possibly, improve the understanding and treatment, of patients afflicted with organically-induced psychiatric conditions. Its implications for clinical practice raise several questions. We hope this report will stimulate renewed interest in this field and although the findings do not contribute to a new conceptual understanding of OBS, they do suggest directions for further research on their management.
